# Quality Control of Stem Cell-Based Cultured Meat According to Specific Differentiation Abilities

**DOI:** 10.3390/cells13020135

**Published:** 2024-01-11

**Authors:** Yuna Naraoka, Yo Mabuchi, Mai Kiuchi, Kyoko Kumagai, Daisuke Hisamatsu, Yosuke Yoneyama, Takanori Takebe, Chihiro Akazawa

**Affiliations:** 1 Intractable Disease Research Center, Juntendo University Graduate School of Medicine, 2-1-1, Hongo, Bunkyo-ku, Tokyo 113-8421, Japan; ynaraoka@juntendo.ac.jp (Y.N.); yo.mabuchi@fujita-hu.ac.jp (Y.M.); m.kiuchi.oo@juntendo.ac.jp (M.K.); k.kumgai.ha@juntendo.ac.jp (K.K.); d.hisamatsu.ap@juntendo.ac.jp (D.H.); 2 Department of Clinical Regenerative Medicine, Fujita Medical Innovation Center, Fujita Health University, 1-1-4, Hanedakuko, Ota-ku, Tokyo 144-0041, Japan; 3 Institute of Research, Tokyo Medical and Dental University, 1-5-45 Yushima, Bunkyo-ku, Tokyo 113-8510, Japan; yone.ior@tmd.ac.jp (Y.Y.); takanori.takebe@cchmc.org (T.T.); 4 Division of Gastroenterology, Hepatology and Nutrition, Cincinnati Children Hospital Medical Center, Cincinnati, OH 45229-3039, USA; 5 Division of Developmental Biology, Cincinnati Children Hospital Medical Center, Cincinnati, OH 45229-3039, USA; 6 Center for Stem Cell and Organoid Medicine (CuSTOM), Cincinnati Children Hospital Medical Center, Cincinnati, OH 45229-3039, USA; 7 Premium Research Institute for Human Metaverse Medicine (WPI-PRIMe), Osaka University, 2-2 Yamadaoka, Suita, Osaka 565-0871, Japan; 8 Department of Genome Biology, Graduate School of Medicine, Osaka University, 2-2 Yamadaoka, Suita, Osaka 565-0871, Japan

**Keywords:** stem cells, bovine cells, culture meat, flow cytometry, adipogenic differentiation

## Abstract

The demand for stem cell-based cultured meat as an alternative protein source is increasing in response to global food scarcity. However, the definition of quality controls, including appropriate growth factors and cell characteristics, remains incomplete. Cluster of differentiation (CD) 29 is ubiquitously expressed in bovine muscle tissue and is a marker of progenitor cells in cultured meat. However, CD29+ cells are naturally heterogeneous, and this quality control issue must be resolved. In this study, the aim was to identify the subpopulation of the CD29+ cell population with potential utility in cultured meat production. The CD29+ cell population exhibited heterogeneity, discernible through the CD44 and CD344 markers. CD29+CD44−CD344− cells displayed the ability for long-term culture, demonstrating high adipogenic potential and substantial lipid droplet accumulation, even within 3D cultures. Conversely, CD29+CD44+ cells exhibited rapid proliferation but were not viable for prolonged culture. Using cells suitable for adipocyte and muscle differentiation, we successfully designed meat buds, especially those rich in fat. Collectively, the identification and comprehension of distinct cell populations within bovine tissues contribute to quality control predictions in meat production. They also aid in establishing a stable and reliable cultured meat production technique.

## 1. Introduction

Global population growth is expected to result in food shortages [1,2], emphasizing the crucial need for a stable alternative protein supply and technological advancements. The conventional approach to meat production, relying on livestock, is environmentally unsuitable due to climate change and the increasing spread of infectious diseases. Therefore, a technological shift in meat cultivation is imperative to ensure a steady meat supply while minimizing environmental impact. Cell extraction, cell culture, and processing are the three main points of production processes for cultured meat. Developing materials such as cultural media, mass culture, and processing methods poses challenges [3,4,5,6]. Additionally, several issues need to be addressed before consumers widely accept cultured meat as a new food product. Consumers base their purchasing decisions on taste, texture, price, and nutritional value. In addition, consumers in different countries emphasize concerns such as adverse effects on the body; these factors vary [7]. Hence, labeling, packaging methods, and marketing strategies for keeping cultured meat fresh are essential considerations. Consumer acceptance of cultured meat varies by age, gender, region of residence, income, and education, but safety concerns are common across all demographics [8]. Therefore, the development of safe cultured meat technology that can meet the needs of many people is essential to supporting the widespread acceptance of cultured meat as a sustainable product.

Recent years have witnessed the emergence of cellular agriculture, aiming to address these challenges by producing cell-based food for sustenance [9,10,11]. Cellular agriculture utilizes stem cell and tissue engineering techniques to produce food historically obtained from livestock. The primary constituent of meat is muscle tissue originating from the musculature of slaughtered animals. Muscle stem cells play a pivotal role in myogenesis in vitro [12]. Choi et al. employed magnetically activated cell sorting (MACS) to isolate purified porcine skeletal muscle-derived cells, revealing the essential role of CD56+ cells in muscle differentiation [13]. Ding et al. employed fluorescence-activated cell sorting (FACS) and investigated cultured CD29+CD56+CD31−CD45− cells derived from bovine muscle tissue. This confirmed the expression of Pax7, a marker of undifferentiated muscle satellite cells [14]. Furthermore, they reported that SB203580, a p38 inhibitor, suppresses the decrease in Pax7 expression, thereby maintaining their undifferentiated status. During muscle regeneration, quiescent satellite cells exhibit heightened levels of Wnt7 and its receptor Fzd7 [15]. They exert critical functions in skeletal muscle hypertrophy by directly activating AKT/mTOR signaling [16]. Wnt7 increases the satellite cell numbers without affecting myoblast differentiation or proliferation [17]. Additionally, Wnt signaling is activated following binding with extracellular matrix (ECM) proteins such as fibronectin, which impedes satellite cell differentiation [17]. Thus, regulating muscle differentiation signaling is pivotal for meat development.

Adipose tissue is an essential component of muscle tissue, contributing flavor, taste, texture, appearance, and nutrition, as fat accounts for 30% of biomass [18,19,20]. Mesenchymal stem/stromal cells (MSCs) are an easily accessible source of cells that have numerous applications in regenerative medicine [21,22]. However, the adipogenic differentiation potential of MSCs depends on their tissue origin, with adipose-derived stem cells (ADSCs) demonstrating heightened adipogenic differentiation potential [23]. Disparities in adipogenesis between beef cattle’s intramuscular and subcutaneous adipose progenitors have been documented [24,25]. The study of bovine adipose stem and progenitor cells revealed that preadipocytes can be collected and characterized as CD26−CD146+CD54+ or CD26−CD146+CD54− [26]. Song et al. reported that porcine ADSCs could be differentiated into adipocytes using insulin, dexamethasone, IBMX, and rosiglitazone [27]. Kahn et al. reported the expression of PLIN1 and FABP4 and the accumulation of lipid droplets in cattle ADSCs, observing their contribution to the flavor of cultured meat. Furthermore, they used bovine ADSCs to develop three-dimensional (3D) meat constructs composed of adipocytes and muscle-derived cells differentiated using seven types of fatty acids, including phytanic acid, erucic acid, and elaidic acid [28]. Similarly, Ouchi et al. reported concentration-dependent lipid accumulation in pluripotent stem cell-derived human liver organoids using fatty acids, such as oleic acid [29]. However, effective quality control methods for cell sources and differentiation in cultured meat production have yet to be established.

In our previous studies, bovine muscle-derived CD29-positive (Ha2/5+ clone) cells were used as stem cells for meat cultivation [30]. These CD29+ cells exhibit proliferative ability, multipotency, and self-aggregation capability, allowing them to form three-dimensional structures integrating both muscle and fat, similar to the original tissue. With the vision of creating a new meat source, we have named them “meat buds”. In this study, our goal was to identify the subpopulation of CD29+ cells based on their myogenic and adipogenic differentiation capacities. Specifically, we used antibodies targeting CD44 and CD344, positive markers of bovine skeletal muscle-derived cells identified in our prior report [30]. CD44 is an adhesion molecule, expressed widely in MSCs, that mediates cell–cell and cell–extracellular matrix interactions [31] and presents type 1 collagen or fibronectin-binding sites [32]. CD44 is involved in the regulation of glucose metabolism in metabolic tissues [33] and contributes to the insulin resistance of skeletal muscle tissue. CD344/Frizzled-4 is a receptor for Wnt signaling and activates canonical (beta-catenin) and non-canonical pathways [34]. Thus, these surface antigens are expected to regulate adhesion and differentiation in CD29+ cells. Profiling the proliferation and differentiation characteristics of these subpopulations will enable the identification of cell subsets suitable for cultured meat design. This fosters the creation of tailored meat buds rich in adipose and skeletal muscle constituents. Moreover, comprehending cell characteristics and predicting cell differentiation potential promises to yield meat quality control insights, ultimately supporting the development of sustainable meat production technologies.

## 2. Materials and Methods

### 2.1. Livestock Tissues

The bovine cheek meat used in this study was sourced from ten two-year-old Japanese black beef cattle (*n* = 10) and obtained from Tokyo Shibaura Zoki Co., Ltd. (Tokyo, Japan).

### 2.2. Cell Preparation

After dissection, the bovine muscle tissues underwent digestion with 2 mg/mL collagenase (FUJIFILM Wako Pure Chemical, Osaka, Japan), 10 mM HEPES (Life Technologies, Carlsbad, CA, USA), 1% penicillin/streptomycin (Life Technologies), and 10 μM DNase I (Sigma-Aldrich, St. Louis, MO, USA) in Dulbecco’s modified Eagle medium (DMEM)-GlutaMAX (Life Technologies) at 37 °C for 1 h with continuous shaking. Subsequently, the liquid fraction was collected, diluted two-fold using Hanks’ balanced salt solution (HBSS; FUJIFILM Wako Pure Chemical), and centrifuged at 800× *g* for 10 min at 20 °C. Then, the supernatant was removed. The cell pellet was resuspended in HBSS and filtered through a 100-μm cell strainer (Corning, Durham, NC, USA).

### 2.3. Antibody Staining and Flow Cytometry

For cell isolation, cells diluted in HBSS were subjected to surface analysis using the following human antibodies: BV421-conjugated anti-CD29 (Ha2/5) (BD Biosciences, Franklin Lakes, NJ, USA), allophycocyanin (APC)-conjugated anti-CD44 (IM7) (BD Biosciences), and PE-conjugated and anti-CD344 (CH3A4A7) (BioLegend, San Diego, CA, USA). Dead cells were detected using propidium iodide staining (P4864-10ML, Sigma-Aldrich). Flow cytometry and sorting were conducted, as previously described, using a FACS Aria II instrument (Becton, Dickinson and Company, Franklin Lakes, NJ, USA); 1 × 10^6^ cells were analyzed, and 10,000 cells were seeded on a 10-cm dish and cultured for the experiment [35].

For cell surface marker analysis, the CD29+ cultured cells in passage 2 were detached using trypsin-EDTA (Thermo Fisher Scientific, Waltham, MA, USA). The cells were then stained with antibodies and analyzed via flow cytometry in the same manner used for cell isolation. Data were analyzed using FlowJo 10.8.1 software (Tree Star, Ashland, OR, USA).

### 2.4. Cell Culture

Isolated bovine CD29+ cells were cultured on fibronectin (Sigma-Aldrich)-coated plastic dishes (Thermo Fisher Scientific). Cells were cultured in DMEM supplemented with GlutaMax (Life Technologies), 20% FBS (Sigma-Aldrich), Penicillin–Streptomycin Mixed Solution (Penicillin 10,000 μg/mL, Streptomycin 10,000 μg/mL, Nacalai Tesque, Kyoto, Japan), and 5 ng/mL basic fibroblast growth factor (ReproCell, Kanagawa, Japan) at 37 °C with 5% CO_2_.

### 2.5. Cell Differentiation

For muscle cell differentiation, 1 × 10^4^ CD29+ cells were seeded in a microscope grass chamber slide (Matsunami Grass, Osaka, Japan) and cultured in DMEM with 20% FBS. After 3 days, the culture medium was changed to a myogenic differentiation medium comprising DMEM with GlutaMax (Life Technologies). It was supplemented with 5% horse serum (Life Technologies), 100 units/mL penicillin, and 100 μg/mL streptomycin (Life Technologies) for 5 days, as previously described [36]. Stained cells were observed using a BZ-X710 microscope (Zeiss, Oberkochen, Germany).

For osteogenic differentiation, 1 × 10^4^ CD29+ cells were seeded in a 24-well plate (Greiner Bio-One, Kremsmünster, Oberösterreich, Austria) and cultured in DMEM with 20% FBS. After 3 days, the culture medium was changed to a human mesenchymal stem cell (hMSC) osteogenic differentiation medium (#PT-3002, Lonza, Basel, Switzerland) and cultured for two weeks, as previously described [37]. The osteoblastic differentiation medium contained dexamethasone, ascorbate, L-glutamine, and B-glycerophosphate. Before staining, the media were aspirated, and cells were washed twice with PBS and fixed at 4% paraformaldehyde at 25 °C for 15 min. Fixative residues were removed by washing with PBS. The cells were incubated with Alizarin Red solution (ARS) (1%, pH 4.0; 011-01192, FUJIFILM Wako Pure Chemical) at 37 °C for 15 min. Subsequently, the ARS solution was discarded, and the cells were washed five times using PBS. Stained cells were observed using a BZ-X710 microscope (Keyence, Osaka, Japan).

For chondrogenic differentiation, 1 × 10^4^ CD29+ cells were seeded in a 24-well plate (Greiner Bio-One, Kremsmünster, Oberösterreich, Austria) and cultured in DMEM with 20% FBS. After 3 days, the culture medium was changed to a hMSC chondrogenic differentiation medium (#PT-3003, Lonza) for two weeks, followed by Toluidine Blue staining [37]. The chondrogenic differentiation medium contained dexamethasone, ascorbate, insulin-transferrin-selenium, pyruvate, proline, L-glutamine, 10 ng/mL of TGF-β3 (BioLegend), and 50 ng/mL of BMP6 (BioLegend). Before staining, the media were aspirated, and cells were washed twice with PBS and fixed at 4% paraformaldehyde at 25 °C for 15 min. Fixative residues were removed by washing with PBS. The cells were washed twice with 300 µL of PBS and stained with a 0.05% toluidine blue solution for 30 min. Stained cells were observed using a BZ-X710 microscope (Keyence). All images were assessed using ImageJ 1.53k software.

After 3 days of seeding cells, we experimented with two methods for adipogenic differentiation. In the first method, CD29+ cells were cultured in a hMSC adipogenic differentiation induction medium (#PT-3004, Lonza) and a hMSC adipogenic maintenance medium (#PT-3102A, Lonza) for 2 weeks according to the manufacturer’s recommendation. In the second method, after 5 days of muscle differentiation, CD29+ cells were cultured in an adipogenic differentiation medium consisting of DMEM with GlutaMax (Life Technologies). They were supplemented with 5% horse serum (Life Technologies), 1 μM IBMX (FUJIFILM Wako Pure Chemical), 1 μM dexamethasone (FUJIFILM Wako Pure Chemical), 1 mM insulin-transferrin-selenium (Thermo Fisher Scientific, Waltham, MA, USA), 50 μM indomethacin (Sigma-Aldrich), and 500 μM oleic acid (Tokyo Chemical Industry, Tokyo, Japan) for 3 days. Subsequently, the medium was replaced with DMEM supplemented with GlutaMax (Life Technologies), 5% horse serum (Life Technologies), 1 μM IBMX, 1 μM dexamethasone, 50 μM indomethacin, and 500 μM oleic acid. Cells were cultured in these two media for 2 weeks, with medium changes occurring every 3 days. Stained cells were observed using a BZ-X710 microscope (Keyence). All chemicals used in the experiment were for research purposes only.

### 2.6. Oil Red O Staining

Cells were fixed using neutral-buffered 4% paraformaldehyde (Nacalai Tesque) for 15 min at 25 °C. Subsequently, cells were washed three times with PBS. Then, the cells were acclimated with 60% 2-propanol (Nacalai Tesque) for 1 min. To stain lipid droplets, cells were treated with an Oil Red O (Muto Pure Chemicals, Tokyo, Japan) solution for 30 min at 25 °C. After washing with 60% 2-propanol for 30 s, the cells were further washed with water three times. Stained cells were observed using a BZ-X710 microscope (Keyence).

### 2.7. Differentiation of CD29+ Cells to Meat Bud Spheroid

Following cell expansion, 1 × 10^5^ CD29+ cells were seeded in a 96-well U-bottom plate (IWAKI, Chiyoda City, Japan) to form spheroids. Subsequently, the plate was centrifuged at 4 × 400× *g* for 3 min. After 3 days of incubation, spheroids were cultured in a myogenic differentiation medium comprising DMEM with GlutaMax (Life Technologies) supplemented with 5% horse serum (Life Technologies) for 5 days. Next, spheroids were cultured in adipogenic differentiation medium comprising DMEM with GlutaMax (Life Technologies), supplemented with 5% horse serum (Life Technologies), 1 μM IBMX, 1 μM dexamethasone, 1 mM insulin-transferrin-selenium, 50 μM indomethacin, and 500 μM oleic acid (Tokyo Chemical Industry) for 3 days. Subsequently, the medium was replaced with DMEM supplemented with GlutaMax (Life Technologies), 5% horse serum (Life Technologies), 1 μM IBMX, 1 μM dexamethasone, 50 μM indomethacin, and 500 μM oleic acid. Cells were cultured for 2 weeks to facilitate adipogenic differentiation. All chemicals used in the experiment were for research purposes only.

### 2.8. Immunofluorescent Staining

CD29^+^ cells in the glass chamber or muscle-sectioned tissue were fixed in 4% paraformaldehyde for 10 min. After washing with PBS, cells were permeabilized with 0.2% Triton-X/PBS for 5 min and blocked with Blocking One solution (Nacalai Tesque) for 1 h. Subsequently, CD29+ cells were stained with monoclonal anti-desmin (mouse IgG, 1:100 dilution, #D1033; Sigma-Aldrich). Muscle sectioned tissue was stained with monoclonal anti-Pax7 (mouse IgG1κ, 1:50 dilution, #sc-81648; Santa Cruz, CA, USA) and APC-conjugated anti-CD29 (Ha2/5) (BD Biosciences). Primary antibodies were incubated with 2% BSA/PBS at 4 °C overnight. Cells or sectioned tissue were washed with PBS twice and stained with Alexa Fluor 594-conjugated anti-mouse IgG1 second antibody (1:1000, Life Technologies), Alexa Fluor 488-conjugated anti-mouse IgG1 second antibody (1:1000, Life Technologies), and Hoechst 33258 (1:1000, Dojindo, Kumamoto, Japan) for nuclear staining. After washing with PBS twice, cells were mounted using PermaFluor™ Adqueous Mounting Medium (Thermo Fisher Scientific) and visualized using a BZX-710 microscope (Keyence, Osaka, Japan).

### 2.9. Whole-Mount Immunofluorescent Staining

CD29+ spheroids were harvested by pipetting and fixed with 4% paraformaldehyde for 24 h. Subsequently, the CD29+ spheroids were washed with PBS, permeabilized using a 0.2% Triton-X/PBS solution for 5 min, and subsequently blocked with the Blocking One solution for 1 h. The same primary antibodies and dilutions, as detailed in the “Immunofluorescence Staining” section, were employed. Subsequently, the CD29+ spheroids were washed with PBS and stained with Hoechst 33258 (1:1000, Dojindo, Kumamoto, Japan) and Alexa Fluor 594-conjugated anti-mouse IgG second antibody (diluted to 1:1000; Life Technologies). After that, cells were stained with BODIPY^®^ 493/503 for lipids (1:2500, Thermo Fisher Scientific) for 30 min at 37 °C, as previously described [29]. The CD29+ spheroids underwent additional washing steps and were mounted using a PermaFluor^TM^ Aqueous Mounting Medium. Ultimately, the stained CD29+ spheroids were visualized and scanned using an LSM 700 confocal microscope (Zeiss).

### 2.10. Colony-Forming Unit Fibroblast Assay

The colony-forming unit fibroblast assay was conducted by culturing 2000 sorted cells on a fibronectin-coated 100-mm dish for 14 days in DMEM-GlutaMax supplemented with 20% FBS (Life Technologies), 1% penicillin/streptomycin, and 5 ng/mL of basic fibroblast growth factor (ReproCell), as previously described [35]. The culture medium was refreshed biweekly. Crystal Violet (1 g) (FUJIFILM Wako Pure Chemical) was diluted in 100 mL of methanol to prepare the Crystal Violet solution. This solution was mixed with an equal volume of 4% paraformaldehyde (PFA). Following filtration, the Crystal Violet/PFA mixture was added to each well, and the dish was incubated for 20 min at 25 °C. Subsequently, cells were rinsed three times with PBS, and colonies were quantified.

### 2.11. RNA Isolation and Quantitative Real-Time PCR

Total RNA was extracted from cells using the RNeasy Plus Mini Kit (Qiagen, Hilden, Germany), following the manufacturer’s protocol. Subsequently, cDNA was synthesized using 100 ng RNA, oligo (dt) primers, and PrimeScript RT Master Mix (Takara, Shiga, Japan). The 100-fold diluted cDNA samples were subjected to quantitative real-time PCR, involving 40 cycles of amplification (95 °C for 15 s, 60 °C for 60 s) using specific forward and reverse primers (10 μM) and Power Track SYBR Green Master Mix (Life Technologies). Amplification was performed on a Quant Studio 5 Real-Time PCR system (Thermo Fisher Scientific). Gene expression levels were normalized to those of β-actin. Each sample was analyzed in duplicate. The comparative Ct method (2^−ΔΔCt^) was applied, where ΔΔCt = ΔCt (sample control) − ΔCt (housekeeping control, *ACTB*) [38]. Primer sequences used for RT-qPCR are provided in Table S1.

### 2.12. Electron Microscopy

Samples were fixed using 2.5% glutaraldehyde (Muto Pure Chemicals). The Laboratory of Morphology and Image Analysis at Juntendo University oversaw sample preparation. After adsorption onto copper grids and subsequent negative staining with 2% uranyl acetate, the samples were evaluated using Transmission Electron Microscopy (JEM-1400Flash, JEOL Ltd., Tokyo, Japan). All images were assessed for oil droplets greater than 4 µm per droplet using ImageJ 1.53k software.

### 2.13. Ststistical Analysis

The data were analyzed for normality using the Shapiro–Wilk test. Unpaired and two-tailed Student’s *t*-tests were employed to compare the two groups for variables with a parametric distribution. For variables with a non-parametric distribution, statistical analysis was performed using the Kruskal–Wallis test with the Bonferroni correction for multiple comparisons. Results are expressed as mean ± SE. Results with * *p* < 0.05 were considered significant.

## 3. Results

### 3.1. CD29+ Cell Fractions Are Composed of Heterogeneous Populations

**Figure 1:**
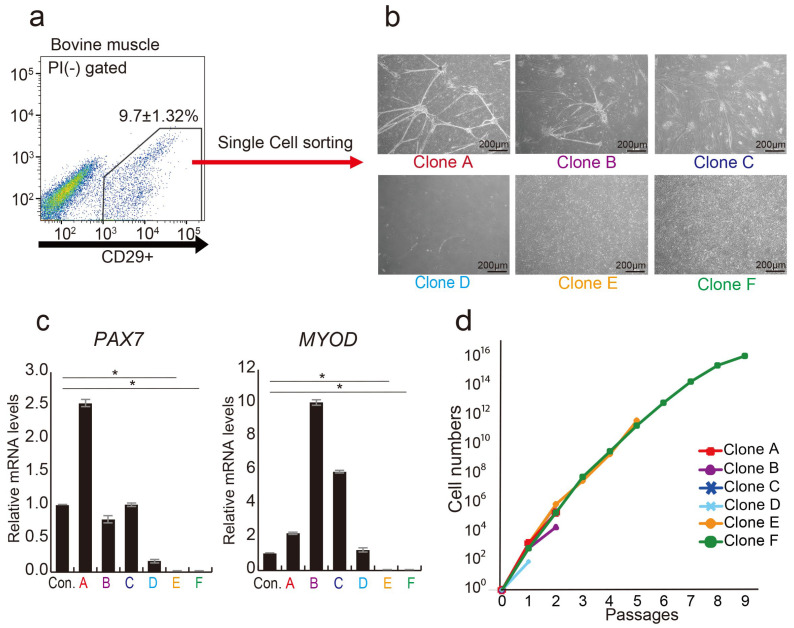
Analysis of CD29+ cells as a heterogeneous population. (**a**) Representative flow cytometric profiles of bovine muscle tissue stained with anti-CD29 antibody. (**b**) Phase-contrast micrographs depicting three distinct colony-forming cell types as myogenic, mesenchymal-like, and adipogenic cell types were isolated by single-cell sorting on day 14: myogenic (Clone A, B, and C), mesenchymal-like (Clone D), and adipogenic (Clone E and F). Scale bar = 200 μm. (**c**) Relative expression levels of myogenic genes *PAX7* and *MYOD* in CD29+ cells were assessed using real-time RT-PCR. CD29(Ha2/5)-positive cells were used as a control. (* *p* < 0.05) (**d**) Numbers of the six cell types were cultured on fibronectin-coated culture dishes until 9 passages.

Previous investigations have demonstrated that CD29+ cell populations in bovine muscle tissue have highly proliferative cells [30]. Through a single-cell analysis of the CD29+ cell population, we identified myoblast-fused myocyte-like cells (Clones A, B, C, and D) showing characteristics of myocytes [39] and mesenchymal-like cells (Clones E and F), meeting the definition of MSC [40] (Figure 1a,b). Gene expression analysis highlighted the presence of myogenic progenitor markers, *PAX7* and *MYOD*, in four clones (A, B, C, and D), but not in E and F cells (Figure 1c). Long-term culture revealed that cells of type E and F proliferated up to passage 10. In contrast, other clones ceased proliferation at passage 3 (Figure 1d). These findings imply that the CD29+ fraction consists of cells expressing myogenic progenitor genes (*PAX7* and *MYOD*), alongside cells lacking these genes but possessing high proliferative potential. Via bovine muscle tissue immunostaining, we verified that CD29+ cells are derived between muscle fibers, as CD29+ cells and Pax7 expression colocalized in the muscle satellite compartment (Figure S2).

### 3.2. Distinct Functional Populations Based on CD44 Expression

**Figure 2:**
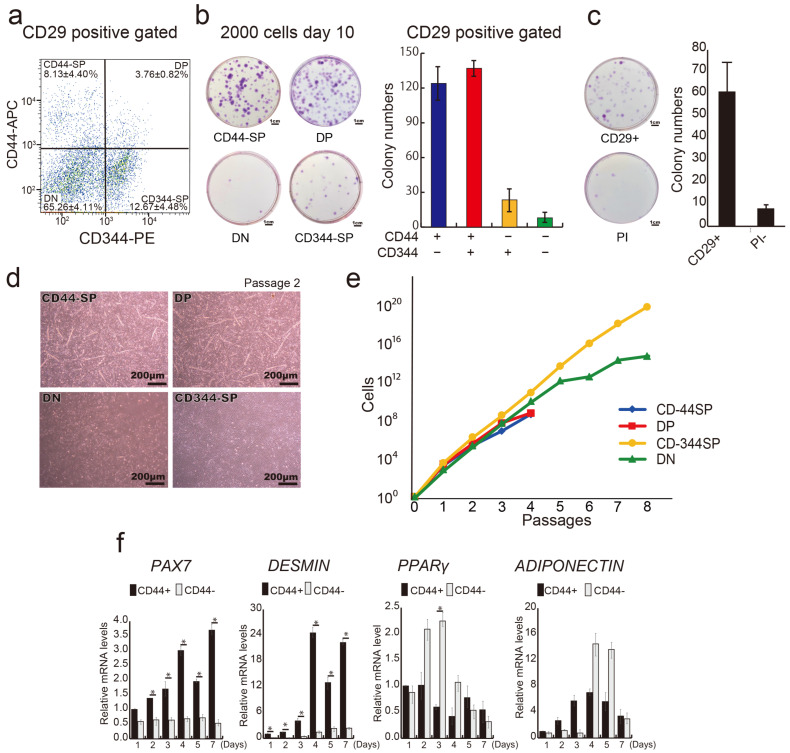
Division of CD29+ cells into distinct groups. (**a**) Representative flow cytometric profiles of CD29+ cells stained with CD44 and CD344-specific antibodies. (**b**) Analysis of colony-forming capacity: 2000 CD44-SP, DP, CD344-SP, and DN cells were sorted and cultured in 10-cm dishes for 10 days, followed by crystal violet staining and colony counting. The number of colonies in each cell population is shown in the graph. (**c**) Analysis of colony-forming capacity: 2000 CD29+ cells were sorted and cultured in 10-cm dishes for 10 days, followed by crystal violet staining and colony counting. The number of colonies in each cell population is shown. PI—propidium iodide. (**d**) Phase-contrast micrographs of CD44-SP, DP, and CD344-SP cells in passage 2 on day 14 under undifferentiated conditions. Scale bar = 200 μm. (**e**) Numbers of CD44-SP, DP, CD344-SP, and DN cells until passage 8 on fibronectin-coated culture dishes. (**f**) Relative mRNA expression of myogenic genes, *PAX7* and *MYOD,* and adipogenic genes, *PPARγ* and *ADIPONECTIN*, in CD44+ or CD44− cells in passage 1 for 7 days. Unpaired and two-tailed Student’s *t*-tests were employed to compare two groups (*n* = 3; * *p* < 0.05).

In our previous report, we demonstrated that anti-CD29 (Ha2/5), anti-CD44 (IM7), and anti-CD344 (CH3A4A7) antibodies effectively enrich positive fractions containing progenitor cells with colony-forming potential [30]. We conducted a comprehensive analysis of bovine tissue cells using these three antibodies. A flow cytometric assessment helped subdivide the CD29+ fractions into four populations: CD44+CD344−fraction (CD44-single positive: CD44-SP) (8.13 ± 4.40%), double positive: DP (3.76 ± 0.82%), CD44−CD344+ fraction (CD344-SP) (12.67 ± 4.48%), and CD44−CD344−fraction (double negative: DN) (65.26 ± 4.11%) (Figure 2a). CD44+ fractions (CD44-SP and -DP) yielded twice as many colonies as those produced by CD29 alone. Conversely, the CD44-negative fraction (CD344-SP and DN) had less than half the colony count for CD29+ cells (Figure 2b,c). After the single-cell sorting of CD44-SP, DP, CD344-SP, and DN into 96-well plates, DP demonstrated the highest colony formation percentage (14.29%) (Figure S1).

Similar to clone A (Figure 1b), myotube-like structures were evident in the CD44-SP and DP groups (Figure 2d and Figure S3). CD44– population (DN and CD344-SP) maintained proliferative capacity up to passage 8, whereas the CD44+ population (CD44-SP and DP) ceased proliferation at passage 4 (Figure 2e). Compared with CD44+ cells (50.6 h), CD44− cells displayed a shorter population doubling time (13.6 h), indicating that CD44− cells were a fast-growing population. Surface antigen analysis at passage 2 confirmed CD44 expression across all populations (Figure S4). CD44+-derived cells displayed elevated expression of CD56, a myogenic marker, during cell culture (Figure S4). Temporal gene expression analysis demonstrated that the CD44+ population had elevated expression of myogenic markers, *PAX7* and *DESMIN*, during culture, whereas the CD44− population exhibited elevated expression of the adipose markers *PPARγ* and *ADIPONECTIN* (Figure 2f). Thus, the CD29+ fraction constitutes functionally distinct cells segregated by CD44 expression, suggesting they could be committed to myocytes or adipose progenitor cells.

### 3.3. Induction of Adipocyte Differentiation Using Bovine Skeletal Muscle-Derived CD29+CD44− Cells

**Figure 3:**
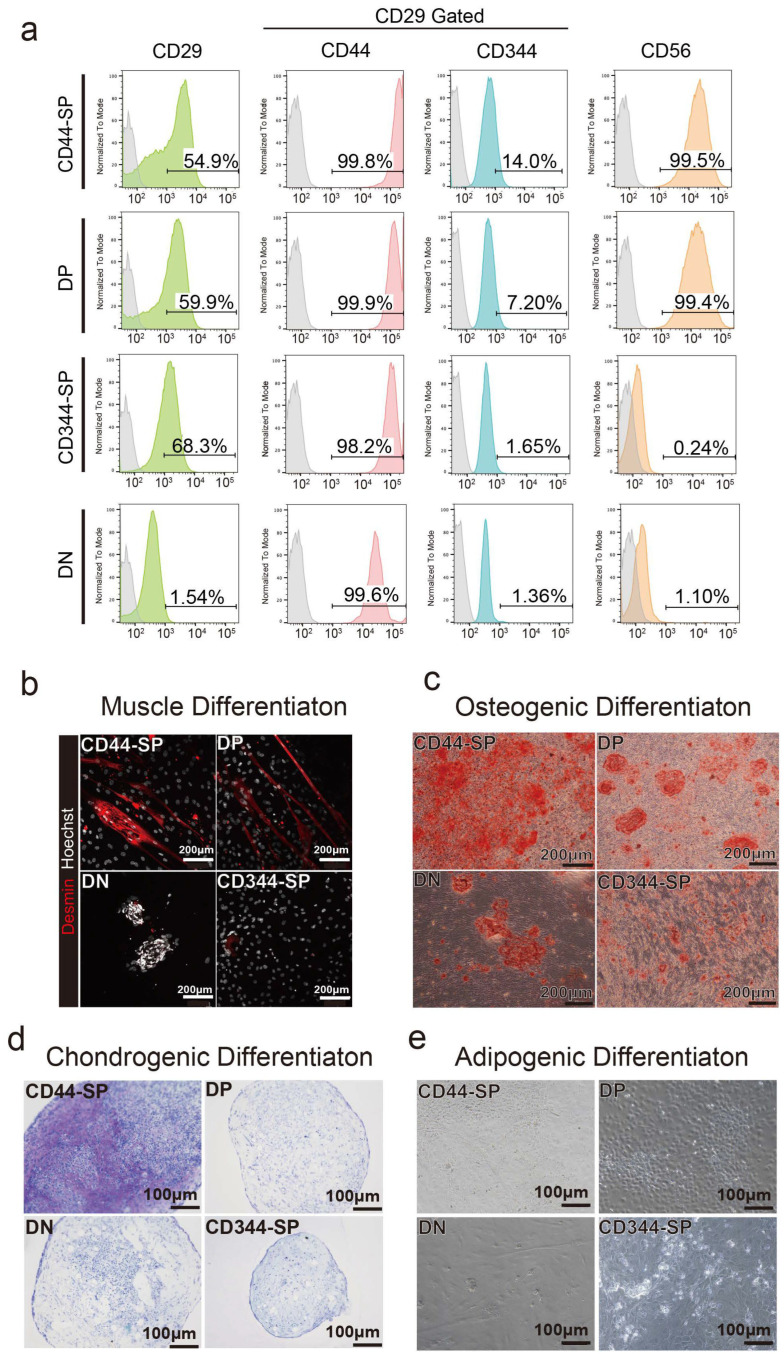
Analysis of cell characteristics. (**a**) Representative flow cytometric profiles when anti-CD29, anti-CD44, anti-CD344, and anti-CD56 antibodies were used for passage 2. (**b**) Myogenic differentiation for 5 days. Desmin is shown in red. Scale bar: 200 μm. (**c**) Alizarin red staining of osteogenic differentiated cells after 2 weeks. Scale bar: 200 μm. (**d**) Toluidine blue staining of chondrogenic differentiated cells for 2 weeks. Scale bar: 100 μm. (**e**) Oil red O staining of adipogenic differentiated cells for 2 weeks. Scale bar: 100 μm.

Surface antigen profiling was performed in undifferentiated states in passage 1. In the DN group, the percentage of CD29+ cells decreased to 1.54%. Over 99% of CD44-SP and DP cells were CD56+, whereas CD344-SP and DN cells exhibited negligible CD56 expression (Figure 3a).

Four CD29+ subpopulations were differentiated to examine their potential for muscle, bone, chondrogenic, and adipogenic differentiation. CD44-SP and DP tended to exhibit demin-positive myotubes (Figure 3b and Figure S5a). The CD44+ cells, especially CD44-SP, presented high osteogenic and chondrogenic differentiation potential (Figure 3c,d and Figure S5a–c). DN cells had a high proliferative capacity, resulting in cells that did not spread but proliferated in clusters (Figure 3b,c). Despite the high adipogenic gene expression in CD344-SP and DN (Figure 2f), the use of commercial adipogenic culture media did not produce adipose differentiation in any fractions (Figure 3d).

**Figure 4:**
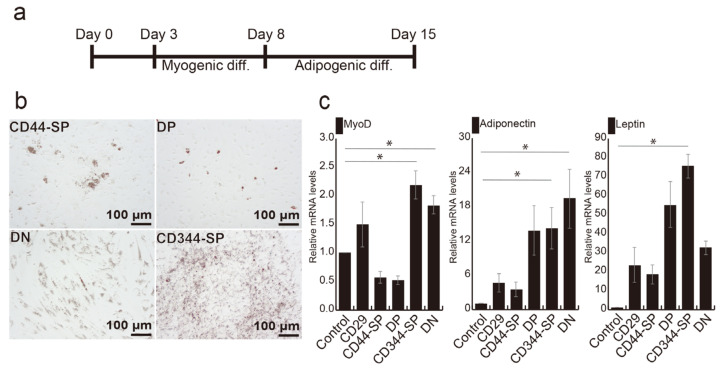
Optimization of adipogenic differentiation. (**a**) Schematic representation of optimized adipogenic differentiation methods. (**b**) Phase-contrast micrographs illustrating adipogenic differentiation using the optimized method. (**c**) Relative mRNA expression of adipogenic and myogenic genes after 7 days of adipogenic differentiation. Undifferentiated CD29+ cells were used as a control sample. The Kruskal–Wallis test with the Bonferroni correction was performed for multiple comparisons (*n* = 4. * *p* < 0.05).

We previously established an effective method for generating bovine adipocytes by combining myogenesis and adipogenesis, as shown in Figure 4a [24]. Following myogenic induction, CD344-SP and DN cells exhibited significantly higher *MYOD* gene expression than the control (*p* < 0.05) (Figure S6). Oil red-O staining revealed lipid droplets in all fractions (Figure 4b). Notably, *LEPTIN* expression in CD344-SP cells (*p* < 0.05), *ADIPONECTIN* (*p* < 0.05), and *MYOD* (*p* < 0.05) expression in both CD344-SP and DN cells were significantly higher compared with those in undifferentiated CD29+ cells (control) (Figure 4c).

### 3.4. Meat-Like Structure Design Via Constitutive Cell Regulation

**Figure 5:**
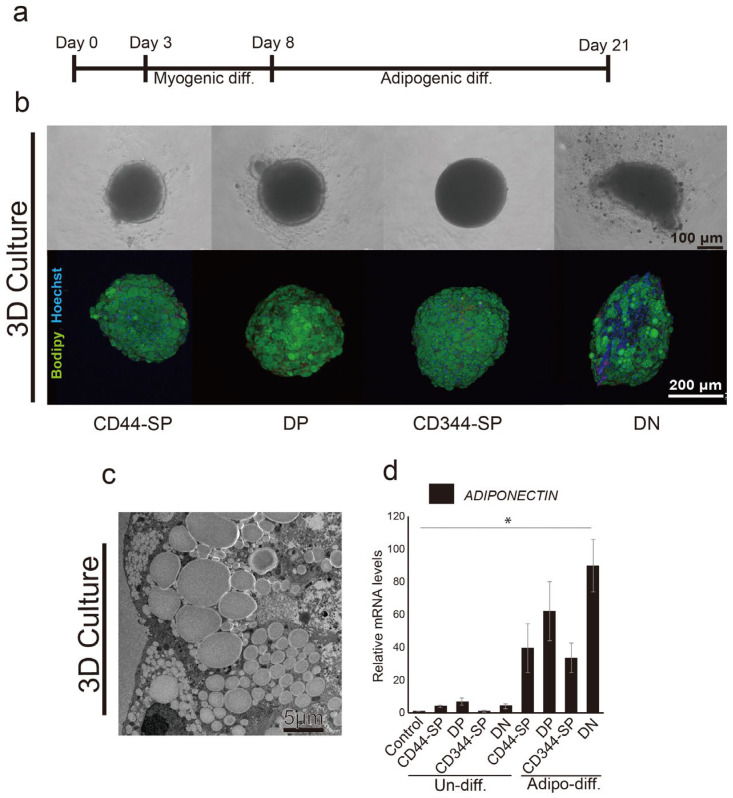
Adipogenic differentiation in 3D culture using the optimized protocol. (**a**) Schematic representation of the optimized adipogenic differentiation methods for meat buds (**b**) Immunohistochemical analysis of induced differentiation in CD44-SP, DP, CD344-SP, and DN cells and phase contrast micrographs of cells. BODIPY (indicating lipid droplets) is shown in green, and Hoechst-stained nuclei are shown in blue. (**c**) Transmission electron microscope observations at 1200× magnification in DN. (**d**) The relative expression of *ADIPONECTIN* in CD44-SP, DP, CD344-SP, and DN was measured using real-time RT-PCR. Undifferentiated CD29+ spheroids were used as a control sample. The Kruskal–Wallis test with the Bonferroni correction was performed for multiple comparisons (*n* = 3, * *p* < 0.05).

CD44-SP, DP, CD344-SP, and DN cells in 96-well U-bottom plates showed spheroid formation after 3D culture (Figure 5b, top). DN spheroids exhibited an oval shape and distinct lipid droplet accumulation (Figure 5b, bottom) using adipogenic differentiation methods (Figure 5a). Transmission electron microscopy revealed the accumulation of lipid droplets inside the meat buds (Figure 5c). Furthermore, similar to 2D, DN exhibited significantly higher (*p* < 0.05) *ADIPONECTIN* gene expression than CD44-SP, DP, and CD344-SP (Figure 5d). In particular, lipid droplet accumulation was observed around the inside of the meat buds (Figure S7). To investigate the potential of the designed meat buds, we created spheroids by mixing four-cell fractions. Upon inducing adipogenesis, the designed meat buds prepared by mixing equal proportions of three types of cells from CD44-SP, DP, CD344-SP, and DN showed increased adipogenic gene expression in the spheroids containing DN (Figure S8).

## 4. Discussion

In this study, we identified a subset of the CD29+ cell populations with a propensity for adipogenic differentiation. Upon purification of CD29+ cells expressing CD44 and CD344, we found that CD29+CD44+ cells demonstrated a heightened proliferation rate but limited long-term culture. Conversely, CD29+CD44− cells exhibited prolonged cultivation, with CD29+CD44−CD344− cells indicating robust adipogenic differentiation, even in 3D culture—a valuable trait for meat cells requiring substantial fat content. These findings hold significance for quality control in cultured meat development.

While we successfully created a meat bud by combining muscle and fat components [30], CD29+ cells represent a heterogeneous population with varying cell characteristics. Therefore, further fractionation of CD29+ cells was attempted to achieve uniform meat buds. Our previous study declared that CD29+ cells were between muscle fibers using Pax7 immunostaining of bovine muscle tissue [30]. In this study, CD29+ cells and Pax7 expression colocalized between muscle fibers (Figure S2). Jankowski et al. reported that most satellite cells derived from avian muscle tissue expressed desmin and MyoD, as well as Pax7 and myogenin, in 17% and 14%, respectively [41]. Myosatellite cells are generally quiescent and undergo remodeling and reconstruction only when necessary [42]. As shown in previous studies, these dormant myosatellite cells have multipotency in mice and humans [43,44]. In the present study, the bovine CD29+ cells were probably satellite cells expressing Pax7 from the basal lamina, which is consistent with previous studies regarding multipotent populations.

Dohmen et al. suggested that bovine skeletal muscle-derived CD29+CD56- cells fractionated using FACS are adipogenic progenitor cells [45] and retained proliferative potential even in extended culture. Furthermore, they reported the expression of genes, including *FABP4* and *ADIPONECTIN*, upon adipogenic differentiation, which is consistent with the characteristics of CD29+CD44− cells in this study. However, CD29+ and CD44+ cells are considered adipose-derived stem cells [37,46]. Song et al. reported that porcine subcutaneous fat-derived stem cells predominantly display the CD29+CD44+ phenotype and differentiate into adipocytes [21]. In avian MSCs, CD29 and CD44 have been identified as surface antigen markers for cells with adipogenic differentiation potential [47,48]. However, previous studies utilized a pre-plate method to isolate cells, raising uncertainties regarding whether muscle-derived CD44+ cells differentiate into adipocytes before culture.

There are several reports of subpopulations in skeletal muscle. In human skeletal muscle, single-cell RNA-seq analysis revealed two subpopulations: a quiescent subpopulation expressing Pax7 and the cyclin-dependent kinase inhibitor, and a subpopulation expressing Myf5, a marker of activated satellite cells with low Pax7 expression [49]. Furthermore, single-cell RNA-seq of bovine skeletal muscle-derived cells also reported that there are cells expressing satellite cell markers such as MYF5, CHODL, NOTCH3, and CHRDL2, and a more quiescent population with low levels of MYF5 and ACTA2 [50]. These reports were similar to the characteristics of CD44+ or CD44- cells in this study, supporting the results of this study. Our previous research also demonstrated that CD44 and CD344-positive cells derived from bovines are proliferative cells capable of forming colonies [30]. Based on these findings, it is plausible to consider that within the population of cells expressing CD44 and CD344 proteins, there is an enrichment of cells capable of in vitro proliferation. Regarding gene expression, CD44 and CD344 are important molecules expressed in various cells [33,51,52]. However, gene expression does not necessarily indicate protein expression, and vice versa.

In this study, we observed that a portion of the CD44− cell population within the muscle converted into CD44+ cells upon culture (Figure 3a and Figure S2). In passage 2, more than 99% of DP and CD344-SP cells converted into CD44+CD344− cells, while only 73% of DN cells converted. During culture, about 44.7% of DN, initially CD29+ in the muscle tissue, converted into CD29- cells (Figure 3a). CD344-SP readily differentiated into adipocytes, aligning with the findings of Dohmen et al. [45]. Conversely, Qian et al. noted that primary mouse and human bone marrow-derived mesenchymal stem/progenitor cells lack CD44 expression [53]. They found that human CD44− cells exhibited 94-fold higher colony-forming capacity than CD44+ cells, with elevated ECM protein gene expression. Moreover, in primary cells, cell cycle analysis indicated that 0.6% of CD44+ cells were in the G0 phase, whereas 15% of CD44− cells were in the G0 phase. CD44+ cells rapidly lost proliferative ability at passage 2, which is consistent with the findings of the present study.

AMP-activated protein kinase (APMPK) is an enzyme composed of a trimer of α, β, and γ subunits. It is involved in cellular energy state homeostasis and activates glucose and fatty acid uptake and oxidation in response to decreased cellular energy [54]. AMPKα1 plays a significant regulatory role in muscle differentiation by enhancing the myogenic differentiation of myoblasts [55]. CD44 regulates energy metabolism by stimulating SRC/AKT/AMPK signaling [56]. The muscle differentiation potential of the CD44+ fraction in this study may be due to AMPKα1 enhancement, and regulating AMPKα1 may support quality control of meat buds.

Wnt signaling molecules are pivotal in myogenic progenitor cell development and myoblast formation [57,58]. CD344/Frizzled4 is a Wnt receptor [34] that is expressed in various tissues [51,52]. Bone marrow-derived MSCs rarely express CD344/Frizzled4 [59]. In our study, approximately 16.43% of CD29+ cells expressed CD344 (Figure 2a). CD344-positive cells present in soft-tissue sarcomas exhibit strong proliferation potential [60], similar to the high proliferation rate of DP cells. Moreover, Wnt ligands binding to Frizzled receptors induce Pax7, MyoD, Myf5, and Myogenin expression via canonical or non-canonical pathways, prompting muscle hypertrophy in humans, mice, and chickens [57,58,61,62,63,64]. C2C12 myoblasts and primary myoblasts isolated from mice can differentiate into adipogenic lineages by blocking the Wnt signaling pathway [65]. However, the relationship between Wnt signaling and myogenic differentiation in cattle remains elusive. Notably, the higher adipogenic differentiation potential of DN may have some essential implications for CD344 negativity in terms of suppressing muscle maturation.

MSCs differentiate into myocytes and adipocytes under MyoD and PPARγ regulation, respectively [66]. Sunadome et al. noted PPARγ expression in both adipocytes and myocytes, with accelerated adipogenesis accompanied by rapid MyoD degradation via a ubiquitin-proteasome system [67]. Moreover, they found that *PPARG* expression remained unaffected in myotubes expressing PPARγ, implying the mutual exclusivity of adipogenesis and myogenesis. In this study, direct adipogenic differentiation post-cell seeding did not induce adipocyte differentiation (Figure 3e). In contrast to direct differentiation methods, adipogenic differentiation follows myogenic differentiation with 5% horse serum-induced mRNA expression of *PPARG* and *MYOD1* (Figure S4). Subsequent adipogenic differentiation with insulin, dexamethasone, indomethacin, and oleic acid yielded mature adipocytes expressing *LEPTIN* and *ADIPONECTIN* mRNAs. Primary skeletal muscle cells from MyoD-deficient mice had significantly lower capacity for osteogenesis and adipogenesis [43]. The essential role of the myogenic differentiation process in adipogenesis highlights the vital role of MyoD in this process, which enabled the optimization of adipogenic differentiation in this study. Downregulation of MyoD expression in the adipose fraction involves cEBP/α acetylation, triggering PPARγ expression and adipogenesis [67]. These findings support the notion that DN cells possess high adipogenic differentiation potential and significant fat content in 3D culture. Hisamatsu et al. found that 3D culture-derived spheroids of CD73+ cells exhibited enhanced cellular characteristics and an upregulated expression of immunomodulatory genes when compared with 2D cells [68]. In the present study, there was a tendency for lipid droplets to accumulate more in CD344-SP than in DN in 2D culture and more in DN than CD344-SP in 3D culture. The results suggest that 3D culture enhances the characteristics of DN, which is an important finding for the design of meat buds.

However, our study has a few limitations: Although we primarily compared CD44+/− and CD344+/− cells within CD29+, CD344-SP and DN demonstrated long-term culture and notably high adipogenic differentiation potential. This result might be because of the presence of adipose progenitor cells among CD44− cells. However, the samples in this study were derived from beef cheeks, which could harbor adipose progenitor cells derived from adipose tissue considering the large amount of fat in its skeletal muscle. Furthermore, we did not investigate the signaling and differentiation mechanisms governing the undifferentiated states of bovine cells. In the future, our goal is to generate various fatty acid-conjugated meat buds for customized meat and food production. These outcomes will facilitate the creation of high-value, cultured meat products.

## 5. Conclusions

Clarifying the cell populations purified using specific surface antigens enabled the identification of the cell characteristics contributing to the development of cultured meat. The CD29+CD44-CD344- fraction had a higher accumulation of oil droplets than the other fractions. In contrast, the CD29+CD44+CD344+ fraction had myogenic differentiation ability. Our innovative technology will facilitate the production of meat buds tailored for optimal skeletal muscle and fat content. The technology also allows the creation of meat buds with nutrients matching disease or individual characteristics. Understanding the characteristics of cells in bovine tissue and predicting their differentiation potential would help regulate meat quality and lead to sustainable meat production technology.

## Data Availability

The datasets for this study are available from the corresponding author upon reasonable request.

## Supplementary Materials

The following supporting information can be downloaded at: https://www.mdpi.com/article/10.3390/cells13020135/s1, Figure S1: Single-cell assay in CD44-SP, DP, CD344-SP, and DN populations. Figure S2: Expression of CD29+ and Pax7 is present between muscle fibers. Muscle marker Pax7 is shown in green. CD29 (red) and cell nuclei (Hoechst, blue) are shown. Arrowheads indicate the localization of Pax7. Figure S3: Immunohistochemical analysis of induced differentiation in CD44-SP, DP, CD344-SP, and DN cells in passage 4 day 5 under undifferentiated conditions. Scale bar = 100. Figure S4: Representative flow cytometric profiles analyzing cell surface markers CD44-SP, DP, CD344-SP, and DN in passage 2, stained with CD44 and CD344 antibodies. Figure S5: Differentiation potential of CD44-SP, DP, CD344-SP, and DN. (a) Muscle, (b) osteogenic, and (c) chondrogenic differentiation. Figure S6: Relative mRNA expression of myogenic gene MYOD and adipogenic gene PPARγ after 5 days of myogenic differentiation. Figure S7: Transmission electron microscope observations at 1200× magnification. Oil droplet areas for CD44-SP, DP, CD344-SP, and DN were compared. The DN group exhibited larger droplets. However, no significant changes were observed in the average area of oil droplets in the DN group. Figure S8: Relative expression of *ADIPONECTIN* in designed meat buds using real-time RT-PCR. Table S1: Primer sequences used for RT-qPCR.
