# Supplementary material for: Quality Control of Stem Cell-Based Cultured Meat According to Specific Differentiation Abilities
Source: Cells. 2024 Jan 11;13(2):135. doi: 10.3390/cells13020135 (PMC10814720; doi:10.3390/cells13020135)
Supplement: Supplementary file 1 [file cells-13-00135-s001.zip › cells-2731511-supplementary.pdf]

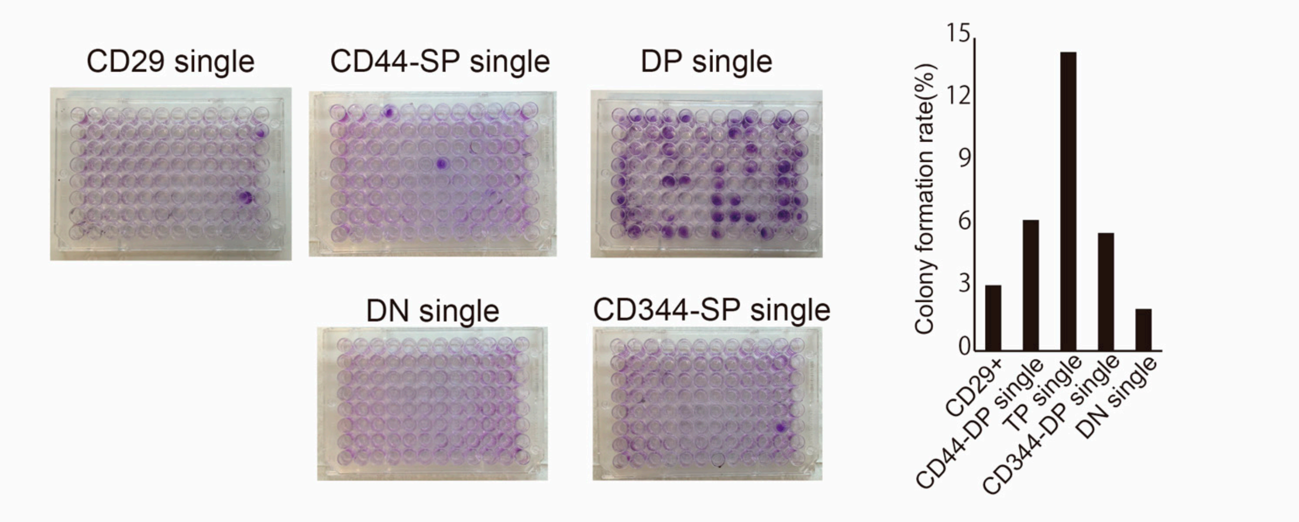

Figure S1. Single-cell assay in CD44-SP, DP, CD344-SP, and DN populations. Single CD44SP, DP, CD344-SP, DN, and CD29+ cells were sorted and cultured in 96-well plates for 2 weeks. Cells were stained with crystal violet, and the colony-forming ratio was calculated based on the number of viable and formed cells. n = 3.

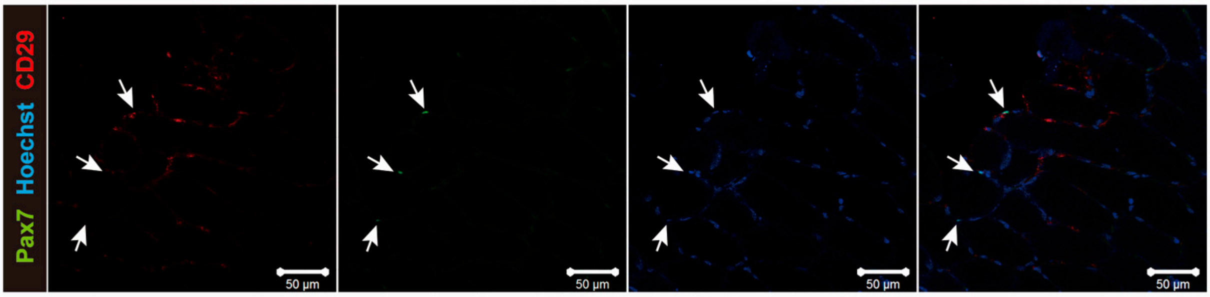

Figure S2. Expression of CD29+ and Pax7 are present between muscle fibers. In bovine muscle tissue immunostaining, we verified that CD29+ cells were derived from between muscle fibers, as CD29 and Pax7 colocalized in the area. Muscle marker: Pax7 is shown in green. CD29 (red) and cell nuclei (Hoechst, blue) are shown. Arrowheads indicate the localization of Pax7.

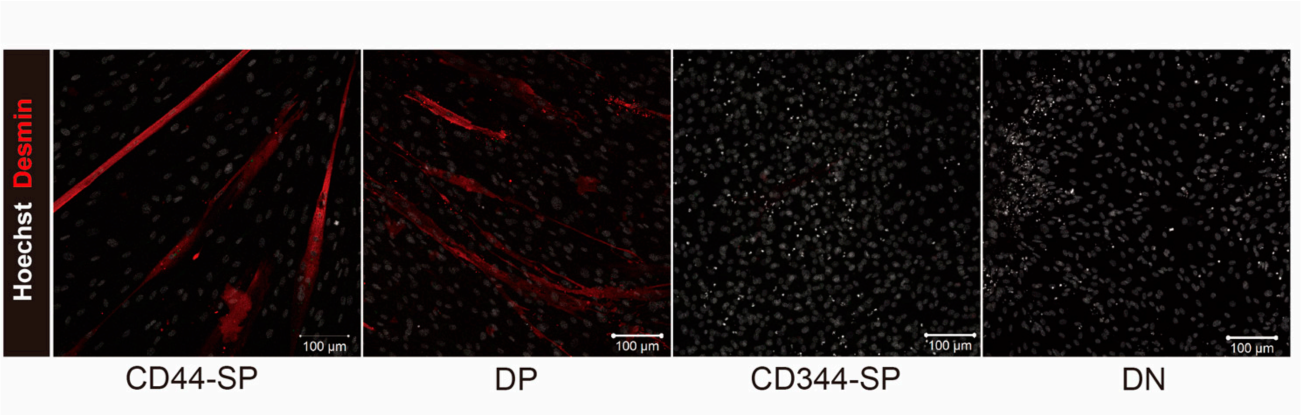

Figure S3. Immunohistochemical analysis of induced differentiation in CD44-SP, DP, CD344-SP, and DN cells, in passage 4 on day 5 under undifferentiated conditions. Scale bar = 100 µm.

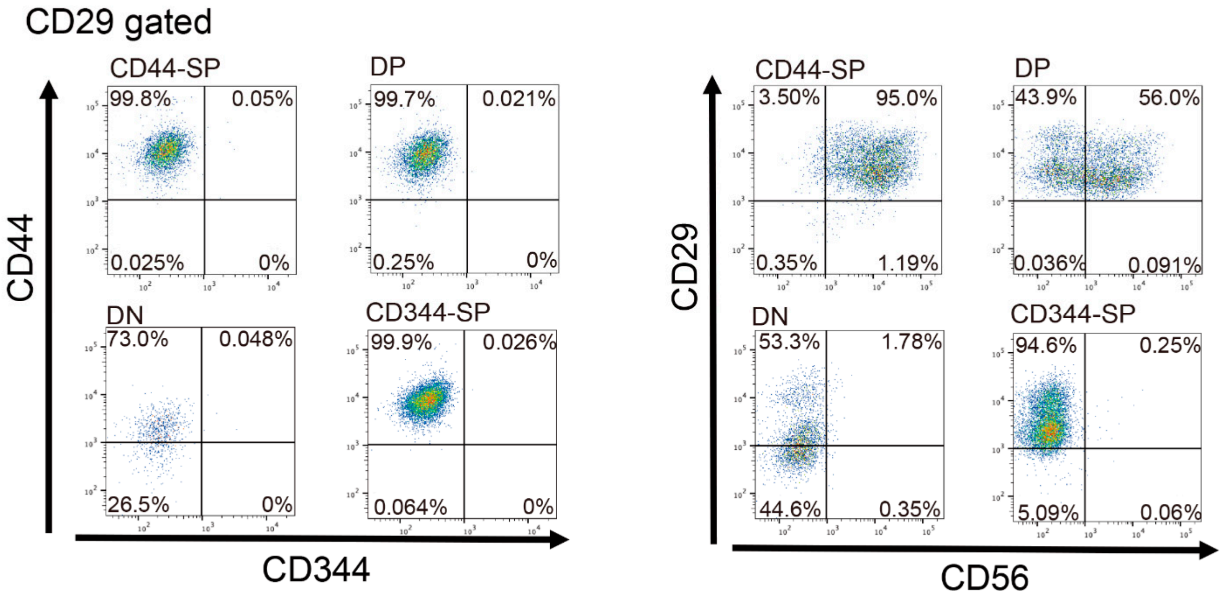

Figure S4. Representative flow cytometric profiles analyzing cell surface markers CD44SP, DP, CD344-SP, and DN in passage 2, stained with CD29, CD44, CD344, and CD56 antibodies.

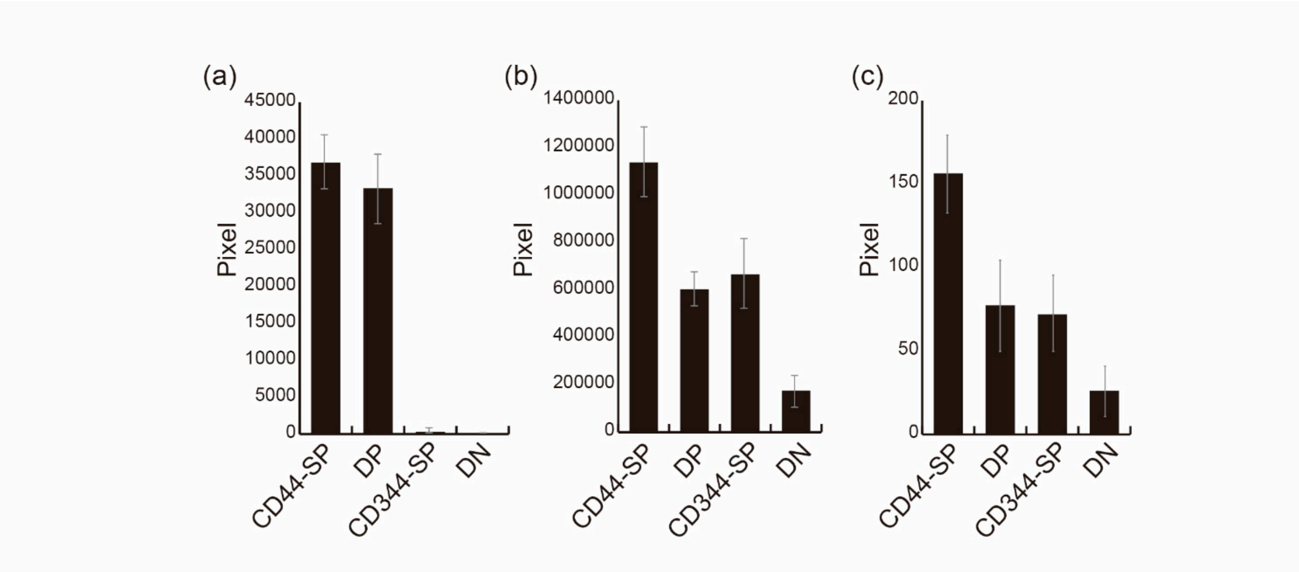

Figure S5. Differentiation potential of CD44-SP, DP, CD344-SP, and DN. (a) Muscle differentiation. (b) Bone differentiation. (c) Chondrogenic differentiation. n = 3

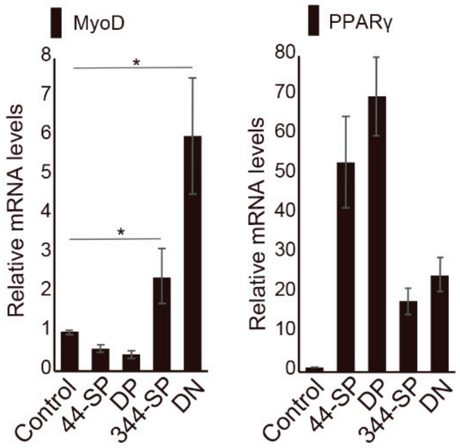

Figure S6. Relative mRNA expression of myogenic gene MYOD and adipogenic gene PPARγ after 5 days of myogenic differentiation. n = 3.

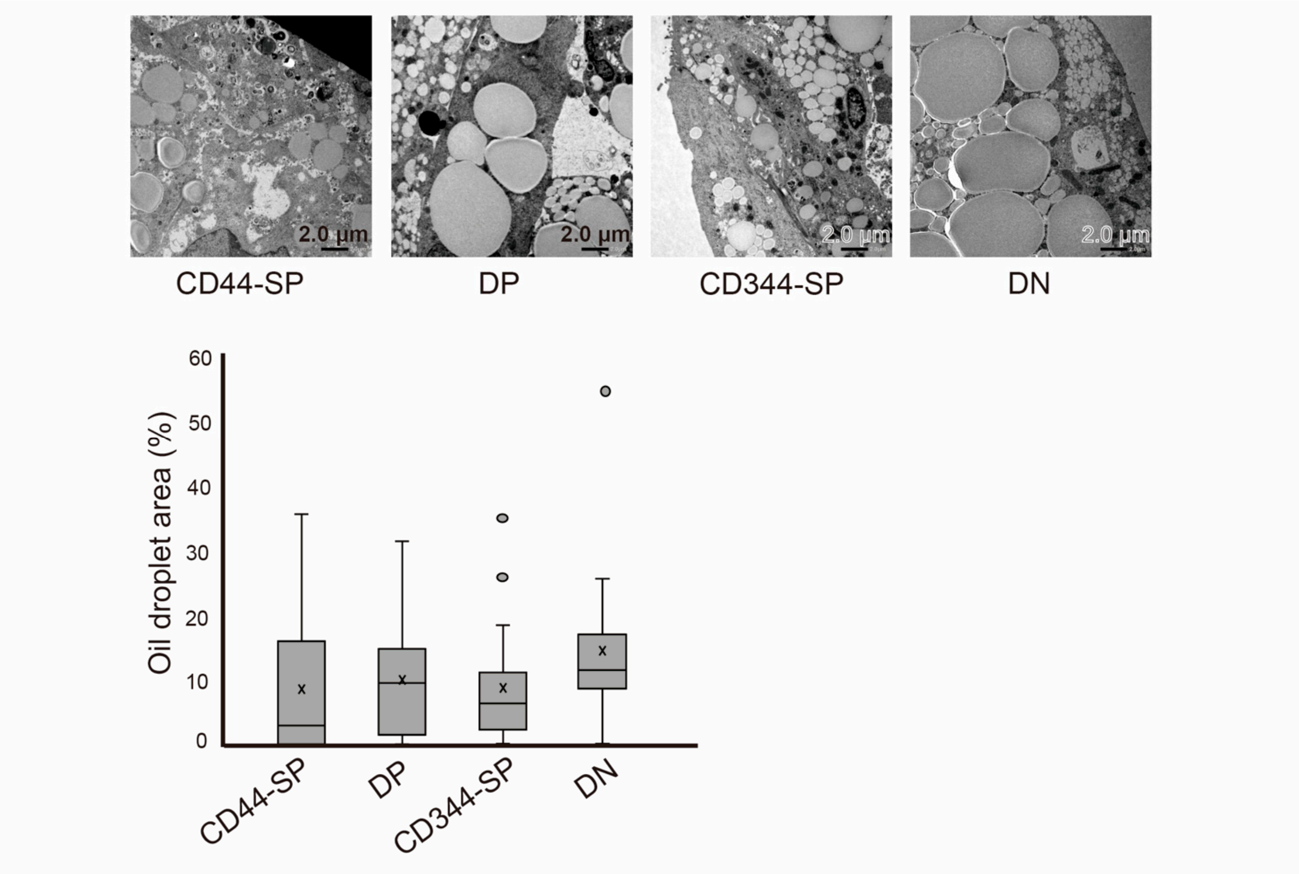

Figure S7. Transmission electron microscope observations at 1200 × magnification. The area of oil droplet volume for CD44-SP, DP, CD344-SP, and DN was compared. DN group exhibited larger droplets. However, no significant changes were observed in the average area of oil droplets accumulated in the DN group.

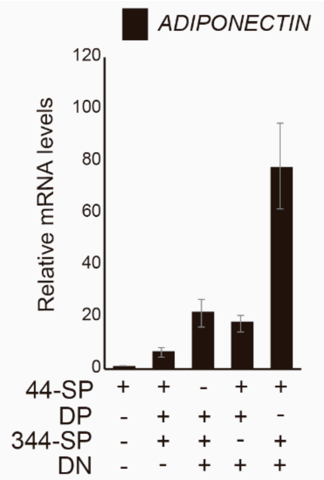

Figure S8. Relative expression of ADIPONECTIN in meat buds prepared by mixing equal proportions of three types of cells from CD44-SP, DP, CD344-SP, and DN was measured using real-time RT-PCR. n = 2.

Supplemental Table S1. Primer sequences for the RT-qPCR.

	5'-Forward-'3	3'-Forward-'5
β-actin	GGCACCCAGCACAATGAAGA	CAGCTAACAGTCCGCCTAGAA
Pax7	GACTCCGGACGTGGAGAAAA	TCCAGACGGTTCCCTTTGTC
Desmin	CGGCTACCAGGACAACATTG	CCGGGTCTCAATGGTCTTGA
C/EBPα	GCAAAGCCAAGAAGTCCG	GGCTCAGTTGTTCCACCCGCTT
C/EBPβ	CGACAGTTGCTCCACCTTCT	CTCGCAGGTCAAGAGCAAG
PPARγ	AAATCCCTGTTCCGTGCTGT	GTCAGCTCTTGGGAACGGAA
Leptin	CGTGACCTTCTTTGGGATTT	AGGGACCATCCACTGAAGTC
Adiponectin	CCTGGTGAGAAGGGTGAGAA	GTGACCTGTCTCTCCAGTCC
